# The analysis of regional ice and snow tourist destinations under back propagation neural network

**DOI:** 10.1016/j.heliyon.2024.e40035

**Published:** 2024-11-01

**Authors:** Fuxue Wang

**Affiliations:** a Tourism and Culture School, the Tourism College of Changchun University, Changchun, 130000, China; b Northeast Asia Research Center on Leisure Economics, Changchun, 130000, China; c Jilin Province Research Center for Cultural Tourism Education and Enterprise Development, Changchun, 130000, China; d Changchun Industry Convergence Research Center of Culture and Tourism, Changchun, 130000, China

**Keywords:** Internet of things, Back propagation neural network, Ice and snow tourism, Spatial-temporal graph convolutional network, Evolutionary characteristics

## Abstract

This study aims to analyze the evolutionary characteristics and development levels of regional ice and snow tourist destinations by integrating the Back Propagation Neural Network (BPNN) within an Internet of Things (IoT) framework. Data from multiple sources are gathered through web scraping technology from various online platforms and are then subjected to cleaning, standardization, and normalization. A feature recognition model for ice and snow tourism is constructed based on a BPNN combined with a Spatial-Temporal Graph Convolutional Network (ST-GCN) algorithm. Experimental results demonstrate that this model excels in convergence speed and prediction accuracy, achieving a final convergence value of 0.059 and a prediction accuracy of 95.74 %, which is at least 4 % higher than that of the baseline BPNN algorithm. Additionally, the model yields Recall and F1 scores of 91.57 % and 89.31 %, respectively. After 98 iterations, the Root Mean Square Error (RMSE) is 6.26, significantly outperforming other model algorithms. These results indicate that the proposed model offers substantial advantages in enhancing the management and service quality of ice and snow tourist destinations, thereby providing valuable technical support and guidance for future intelligent tourism management.

## 1 Introduction

### 1.1 Research background and motivations

In recent years, the rapid advancement of Internet of Things (IoT) technology has facilitated its widespread adoption across various industries. In the tourism sector, IoT technology significantly improves management and service quality through enhanced data collection, real-time monitoring, and intelligent analysis [[1], [2], [3]]. Ice and snow tourism (IST), characterized by its unique experiences and diverse activities, has become increasingly popular, drawing numerous visitors [[4], [5], [6], [7]]. The development of ice and snow tourist destinations is crucial for regional economic growth [8]. However, the evolution and development levels of these destinations are influenced by various factors. Traditional research methods often rely on statistical analyses and empirical judgments, which may not fully uncover the underlying patterns within the data. This highlights the need for a more systematic approach to analysis and research.

Although existing literature extensively covers the application of IoT technology in tourism management—particularly in enhancing visitor experiences and optimizing resource management—most studies focus on specific aspects such as management, marketing, and services. There is a lack of comprehensive analysis addressing the overall evolutionary characteristics and development levels of tourism destinations. The Back Propagation Neural Network (BPNN), a well-established Artificial Neural Network (ANN) model, is renowned for its effective data analysis and prediction capabilities due to its powerful nonlinear mapping, self-learning, and adaptive features [9,10]. For instance, Yağ and Altan (2022) developed an AI-based hybrid algorithm incorporating deep learning techniques to detect plant diseases in agricultural settings in real time, demonstrating AI's efficacy in data analysis and prediction [11]. Recently, there has been increasing research on the application of BPNN for tourism forecasting, market analysis, and user behavior analysis, showcasing its substantial potential for managing complex tourism data [12,13]. Despite its proven performance in data analysis and prediction, the application of BPNN in the context of snow and ice tourism remains relatively underexplored.

Integrating IoT technology with BPNN enables a more nuanced understanding of the evolutionary characteristics and development levels of ice and snow tourist destinations through advanced big data analysis and intelligent prediction. This integration offers valuable scientific evidence for decision-makers.

### 1.2 Research objectives

This study seeks to investigate the evolutionary patterns of regional ice and snow tourist destinations and evaluate their development levels by combining IoT technology with BPNN. The objective is to provide robust data support and decision-making references that enhance the competitiveness of these destinations. IoT technology is employed to gather multidimensional data on these destinations, and the BPNN model is used to analyze their evolution characteristics. Subsequently, this study aims to develop an evaluation model based on BPNN to quantify the development levels of various regional ice and snow tourist destinations. This approach offers scientific evidence for governmental and business planning and management, promotes sustainable regional economic development, and provides valuable insights for practical decision-making. The study's contribution lies in the collection of real-time, multidimensional data and the application of in-depth analysis using the BPNN model. This study not only supports governmental and business planning and management but also highlights the potential of deep learning technologies in the tourism sector, offering new avenues for future research and practice in tourism.

The contributions of this study are as follows.

➢ Model Innovation: This study proposes an integrated model combining BPNN and Spatial-Temporal Graph Convolutional Network (ST-GCN) to analyze and predict the evolutionary characteristics and development levels of IST destinations. This is the first application of such a model in existing research.
➢ Technological Integration: By combining traditional neural network techniques with the emerging ST-GCN technology, this study achieves a technological innovation in the analysis of IST data, enhancing the accuracy and efficiency of predictions.
➢ Data-Driven Decision Support: The study provides a data-driven approach to help tourism managers and decision-makers better understand the development trends of tourism destinations, enabling more scientific and rational management decisions.
➢ Practical Application Guidance: In addition to theoretical model construction and validation, this study explores the model's application in practical tourism management, offering specific technical guidance for the intelligent management and service of IST destinations.

The remainder of this study is organized as follows: The literature review section delves into existing research, providing the theoretical and empirical foundation for this study. The methodology section details the construction of the integrated BPNN and ST-GCN model, including key technical steps such as data collection, preprocessing, model design, and algorithm implementation. The experimental design and performance evaluation section presents the model's performance under controlled experimental conditions, validating its superiority through comparisons with existing algorithms. The model application and discussion section shifts the focus to practical applications, exploring specific case studies of the model in tourism management and discussing potential challenges during implementation. Finally, the conclusion and future work section summarizes the main findings of the research and outlines prospects for future studies. The clear organizational structure of the study not only showcases the findings but also provides valuable references and insights for research and practice in the field of IST.

## 2 Literature review

The IoT facilitates the sensing, identification, and management of diverse information from the physical world by connecting various physical devices and sensors to the internet. The rapid advancement of IoT technology provides robust technical support for the digital transformation of the tourism industry. Extensive research has been conducted on its applications in tourism management, marketing, and services. Tiwari et al. (2022) [14] investigated IoT applications as a sustainable energy management solution in Indian tourist destinations, highlighting its role in promoting sustainable tourism development by enhancing energy efficiency and reducing carbon emissions. Aliyah et al. (2023) [15] conducted a comprehensive study on the impact of artificial intelligence and IoT on smart tourism destinations, emphasizing their pivotal roles in improving visitor experiences and optimizing resource management. Kong (2023) [16] developed real-time processing systems and IoT applications for the cultural tourism sector, advancing cultural tourism by enhancing data processing efficiency and visitor interaction. Li (2023) [17] examined the integration of big data and IoT in cultural tourism and rural revitalization, demonstrating the potential of these technologies to drive rural tourism and economic development. Ma (2024) [18] created an intelligent tourism service system based on IoT and machine learning, improving tourism management and visitor satisfaction through intelligent data analysis and service optimization. Rosário and Dias (2024) [19] reviewed IoT applications in smart tourism, outlining current research trends and future development directions. Zhang (2024) [20] analyzed ecological safety and tourist satisfaction in IST using deep learning and IoT, illustrating the potential of IoT technology in enhancing the IST experience and ensuring ecological safety.

Research on addressing complex second-order boundary value problems has seen significant developments. Arqub and Abo-Hammour (2014) [21] introduced a continuous genetic algorithm that effectively provides numerical solutions for such problems. This approach, further demonstrated by Abu Arqub et al. (2012) [22], optimizes numerical models to ensure accurate predictions in complex model analyses, particularly in addressing boundary value problems with singularities.

The BPNN, a type of multilayer feedforward neural network, has been extensively utilized in data analysis and prediction [23]. For instance, in the financial sector, Li et al. (2023) [24] employed an optimized BPNN model to predict risks in the financial management of listed companies, demonstrating the model's high efficiency in risk identification within the digital economy context. Yang et al. (2023) [25] used BPNN to analyze the spatial spillover effects of technological innovation driven by digital finance, revealing significant spatial correlations in innovation levels across different regions. Liu and Yang (2024) [26] integrated BPNN with rough set theory to develop a financial risk early warning model for listed companies, showcasing its accuracy and reliability in financial risk detection and warning.

In the medical field, Hamoud et al. (2023) [27] proposed a video blood pressure estimation method combining multiple neural network models, achieving excellent accuracy and stability in blood pressure estimation. Selvarajan et al. (2023) [28] conducted a comparative study on various ANN for recognizing the excretory system in medical applications, highlighting significant advantages in precise recognition and classification for specific networks. Vikraman and Jabeena (2024) [29] investigated an optimized Convolutional Neural Network (CNN) for brain MRI image segmentation and compression, significantly enhancing image compression efficiency while retaining essential medical information.

In engineering, Jin et al. (2023) [30] applied an enhanced BPNN for diagnosing power transformer faults, significantly improving diagnostic accuracy. Similarly, Zhou et al. (2023) [31] utilized a BPNN optimization algorithm for fault location in distribution networks, markedly increasing fault location efficiency.

In recent years, the application of BPNN in tourism research has been on the rise, encompassing areas such as tourism demand forecasting, visitor behavior analysis, and tourism satisfaction evaluation. Movahedi et al. (2023) [32] examined the relationship between urban travel demand and electricity consumption through deep learning methods, offering scientific insights and novel approaches for urban planning and tourism resource allocation. Li et al. (2023) [33] enhanced short-term demand forecasting for shared bikes by employing an irregular CNN, thereby improving the understanding of shared bike demand fluctuations. Ma and Liu (2024) [34] utilized a combined deep learning model for forecasting shared bike demand. Liang et al. (2024) [35] developed a hybrid forecasting framework that integrates search engine data and spatial effects to predict aviation passenger demand. These studies provide substantial support for tourism-related decision-making.

In summary, while the applications of IoT and BPNN across various fields have been extensively researched, several challenges and limitations persist. The widespread deployment of IoT devices raises concerns regarding data privacy and security, as the extensive network of sensors and devices can potentially expose sensitive information and increase the risk of data breaches. Furthermore, interoperability and standardization issues among devices pose significant challenges, as compatibility problems between different manufacturers and platforms can limit system scalability and flexibility. Despite its effectiveness in managing complex data and making predictions, BPNN also faces limitations. These models often require substantial amounts of training data, and the training process can be time-consuming. Additionally, BPNN models are prone to overfitting, particularly when dealing with high-dimensional data features, which can adversely affect their generalization ability and predictive performance on new data.

In the realm of snow and ice tourism, selecting an appropriate neural network model is essential for enhancing prediction accuracy and managing complex data. BPNN, a classic multilayer feedforward neural network, is favored for its robust nonlinear mapping capabilities and self-learning features. However, BPNN's capacity to handle spatiotemporal data is limited, which can impede the analysis of tourist behavior and the evolutionary characteristics of tourism destinations. In contrast, the ST-GCN, which integrates Graph Convolutional Network (GCN) and Temporal Convolutional Network (TCN), effectively processes both spatial and temporal dimensions of data. This model has demonstrated exceptional performance in tasks with spatiotemporal dependencies, such as traffic flow prediction and dynamic social network analysis. This study's innovation lies in the integration of IoT technology with BPNN and the incorporation of ST-GCN to systematically analyze the evolutionary characteristics and development levels of regional snow and ice tourism destinations. By utilizing IoT technology to collect real-time multidimensional data and performing in-depth analysis with the BPNN model, the study aims to provide scientific evidence to support governmental and business planning and management of ice and snow tourist destinations.

## 3 Research model

### 3.1 Intelligent demand analysis for IST supported by IoT

**Fig. 1:**
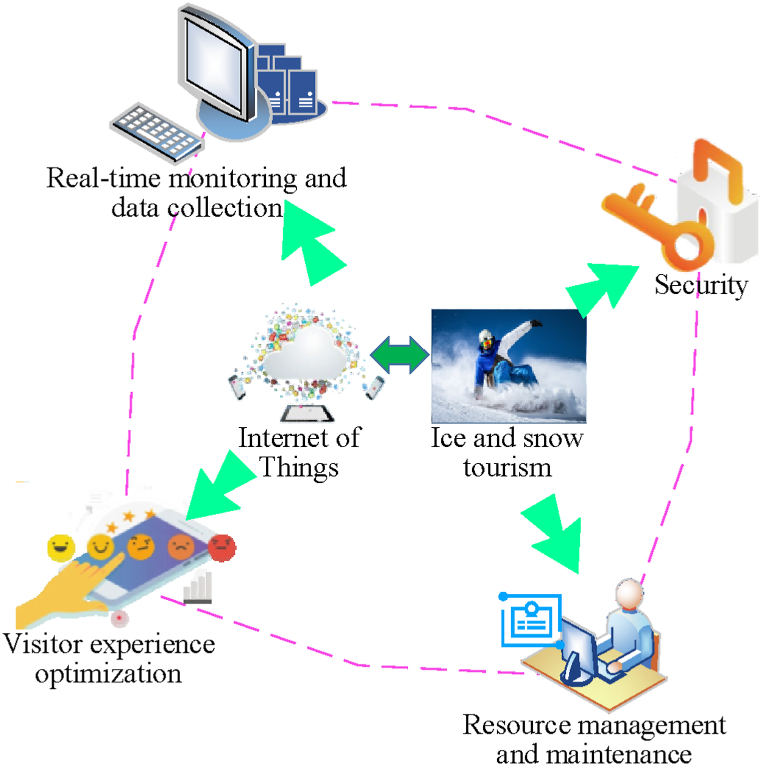
Diagram of intelligent demand in IST supported by IoT.

Ice and snow tourist destinations are locations that leverage ice and snow resources as primary attractions, offering activities such as skiing, ice entertainment, and ice sculpture viewing [36]. These destinations encompass both naturally advantageous ski resorts and man-made ice and snow recreational facilities. Snow and ice tourism emphasizes these ice and snow resources, providing a diverse range of activities and contributing significantly to regional economic development. The advent of IoT technology has profoundly transformed IST by elevating the intelligence level of these destinations [[37], [38], [39]]. IoT technology enhances operational efficiency and service quality through real-time monitoring, visitor experience optimization, safety assurance, and resource management. This advancement not only improves the efficiency and quality of operations but also offers visitors more personalized and secure travel experiences. Fig. 1 depicts the intelligent demands of IST supported by IoT technology.

Fig. 1 illustrates the analysis of intelligent demands in snow and ice tourism through IoT technology, emphasizing its role in advancing smart management and services. IoT technology facilitates comprehensive intelligent management and optimization of services at snow and ice tourism destinations. Firstly, it supports real-time monitoring by collecting and analyzing data on the operational status of ski resorts, snow trails, cable cars, and other facilities, alongside weather and environmental conditions. This capability enables timely alerts for potential safety hazards, thereby enhancing visitor safety. Secondly, IoT technology enriches the visitor experience through the use of smart wearable devices and mobile applications. These tools provide real-time information on attractions, navigation services, and personalized recommendations, thereby improving convenience and satisfaction for visitors. Additionally, IoT enhances safety measures by employing sensors and monitoring devices for continuous surveillance, allowing for the prompt detection and management of emergencies. Finally, IoT technology contributes to resource management by analyzing data related to visitor flow, facility usage, and energy consumption. This analysis supports optimized resource allocation, improved operational efficiency, and the promotion of sustainable development. These analyses of intelligent demands enhance both the management efficiency and visitor experience at snow and ice tourism destinations while providing a robust technological foundation for their sustainable development. By leveraging IoT technology, valuable data and insights are gathered, which support effective feature identification and development level assessment. Furthermore, the integration of BPNN with the ST-GCN algorithm allows for the extraction of actionable information from extensive datasets. This combination facilitates precise analysis and prediction of the evolution characteristics and development levels of ice and snow tourist destinations, thereby advancing their strategic management and planning.

### 3.2 Analysis of IST Feature Recognition Model Based on BPNN integrated with ST-GCN algorithm

**Fig. 2:**
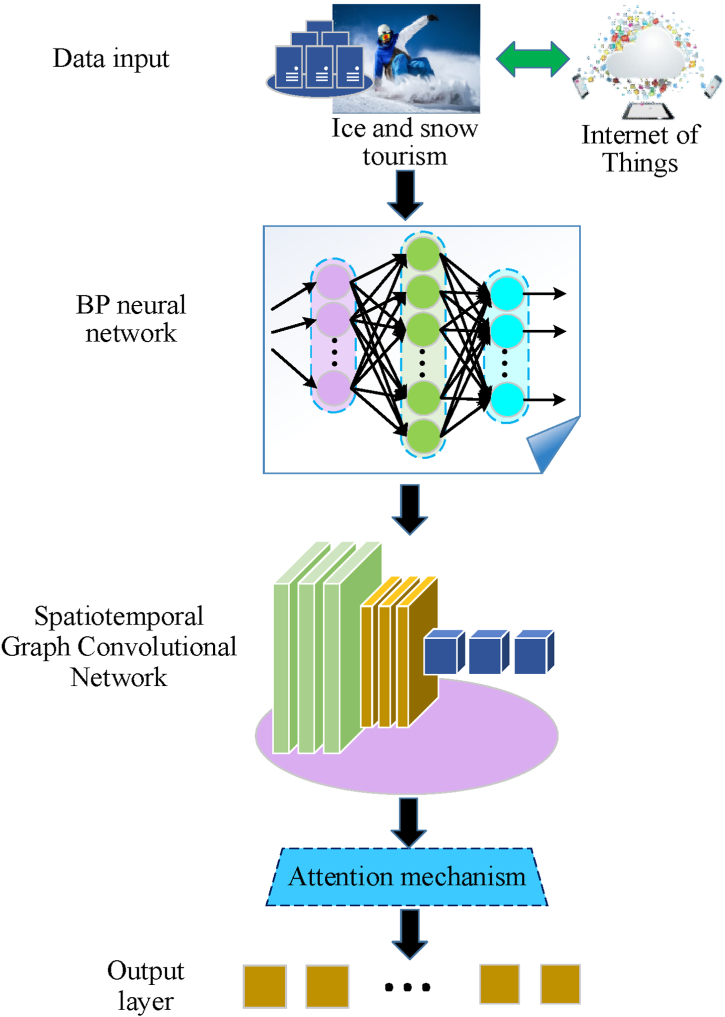
Framework diagram of the IST feature recognition model based on BPNN integrated with ST-GCN algorithm.

The evolution of ice and snow tourist destinations is influenced by visitor behavior and preferences, making it essential to analyze historical travel patterns and performance characteristics. Visitor behavior encompasses spatial movements and durations, which involve multidimensional data, including temporal, spatial, and semantic aspects. Analyzing such behavior necessitates a consideration of both spatial and temporal dimensions. To address this, this study employs the BPNN [[40], [41], [42], [43]] to identify complex patterns and features within IST data. Additionally, the study integrates the ST-GCN [[44], [45], [46]], which processes spatial and temporal information concurrently. The ST-GCN merges Graph Convolutional Networks (GCN) with TCN to handle spatiotemporal dependencies more effectively, making it suitable for tasks such as traffic flow prediction, video analysis, and social network dynamics. The adaptability of ST-GCN to various graph structures and time series lengths enhances its generalization capabilities. Furthermore, its modular design allows for straightforward extension and customization for specific application scenarios, increasing its practicality. Fig. 2 depicts the framework of the IST feature recognition model, incorporating the BPNN and ST-GCN algorithm.

Fig. 2 illustrates a framework for a snow tourism feature recognition model that integrates the ST-GCN algorithm with BPNN. This model is designed to accurately identify and predict the evolutionary characteristics and development levels of snow tourism destinations by leveraging the nonlinear mapping capabilities of BPNN alongside the ST-GCN's proficiency in handling spatiotemporal data. The model begins by processing various input features from snow tourism destinations, such as climate data and visitor flow data. BPNN addresses nonlinear relationships through its hierarchical structure, mapping input features into a high-dimensional space to reveal complex patterns within the data. This is achieved via multiple layers of neurons, each with weights that are iteratively adjusted using the backpropagation algorithm, allowing the network to effectively “learn” the most representative features. Following this, the ST-GCN processes relational data by analyzing interactions between different nodes, such as tourist attractions, which is essential for understanding regional tourism patterns. The TCN component within the ST-GCN captures the evolution of these patterns over time, providing a temporal perspective necessary for predicting future trends. Additionally, an attention mechanism is incorporated into the model to enhance the recognition of key information in the input sequence. This mechanism adjusts the weights of different positions through a parameterized weight matrix, improving the model's ability to adapt to various input sequences. Through this multi-level, multi-angle approach to feature extraction and integration, the model aims to accurately identify the key characteristics of snow tourism destinations, thereby offering scientific decision support for their intelligent and sustainable development.

The optimization of the BPNN involves adjusting weights and biases through the backpropagation algorithm, enabling the model to converge to an optimal state. The output of the BPNN is represented as shown in Equation (1).

(1) $$y=f\left(\sum_{i=1}^{n}w_{i}x_{i}-\theta \right)$$

In Equation (1), y refers to the output, $x_{i}$ refers to the input feature, $w_{i}$ refers to the weight, θ refers to the bias term, and f(⋅) refers to the bias term, and refers to the activation function.

Next, the ST-GCN model further extracts spatial and temporal features from the data. The spatial dimension is processed using the GCN, as shown in Equation (2).

(2) $$H^{\left(l+1\right)}=\sigma \left(D^{-1/2}AD^{-1/2}H^{\left(l\right)}W^{\left(l\right)}\right)$$

$H^{\left(l\right)}$ refers to the node feature matrix of layer *l*, A denotes the adjacency matrix, *D* is the degree matrix, $W^{\left(l\right)}$ represents the weight matrix of layer *l*, and *σ* indicates the activation function. The temporal dimension is handled through the TCN, capturing temporal variations in the data.

The attention layer plays a crucial role by employing a set of parameterized weight matrices to identify and emphasize significant information from the hidden layer output vectors. This mechanism enhances the model's ability to discern the relevance of each position within the sequence, thereby improving its understanding of the sequence's structure. The attention layer dynamically adjusts the weights assigned to each position, allowing the model to effectively accommodate variations in different input sequences.

For a given feature vector *X* related to IST data, the attention mechanism extracts feature information pertinent to a specific task *Q*. Typically, this task *Q* can be represented by a query vector *q*, as depicted in Equation (3).

(3) $$r=Attention\left(q,X\right)=\sum_{i=1}^{N}\alpha_{i}x_{i}$$

r refers to the output feature vector of the attention mechanism, and $\alpha_{i}$ denotes the weight coefficient of the input feature vector $x_{i}$.

$\alpha_{i}$, also known as the probability distribution, expresses the relationship between the input feature vector $x_{i}$ and the query vector *q*. Its result can indicate the importance or relevance of the input feature vector to the query vector, as shown in Equation (4).

(4) $$\alpha_{i}=soft\;\max\left(s\left(q,x_{i}\right)\right)=\frac{\exp\left(s\left(q,x_{i}\right)\right)}{\sum_{i=1}^{N}\;\exp\left(s\left(q,x_{i}\right)\right)}$$

$s\left(q,x_{i}\right)$ refers to the attention scoring function, used to measure the correlation between the query vector *q* and each element in the sequence. The specific implementation depends on the task requirements. Common scoring function models include additive models, dot product models, and bilinear models, which are respectively represented by Equations (5), (6), (7).

(5) $$s\left(q,x_{i}\right)=v^{T}\;\tanh\left(Wx_{i}+Uq\right)$$

(6) $$s\left(q,x_{i}\right)=x_{i}^{T}q$$

(7) $$s\left(q,x_{i}\right)=x_{i}^{T}Wq$$

tanh refers to the hyperbolic tangent function. Vector *v*, matrices *W* and *U* are all model parameters to be learned.

Ultimately, the text information output with weights is obtained, as shown in Equations (8), (9), (10).

(8) $$s=\sum_{t=1}^{k}\alpha_{t}h_{t}$$

(9) $$\alpha_{t}=\frac{\exp\left(e_{t}\right)}{\sum_{i=1}^{n}e_{ti}}$$

(10) $$e_{t}=V_{t}\;\tanh\left(W_{t}h_{t}+b_{t}\right)$$

$e_{t}$ denotes the attention weights obtained for the hidden state $h_{t}$ at time step *t*. $V_{t}$ and $W_{t}$ serve as the weight matrices at time t. $b_{t}$ refers to the bias vector. $\alpha_{t}$ represents the weights normalized using softmax, *i* denotes the number of categories for classification, and s indicates the final feature vector output after weighting with attention weights.

**Fig. 3:**
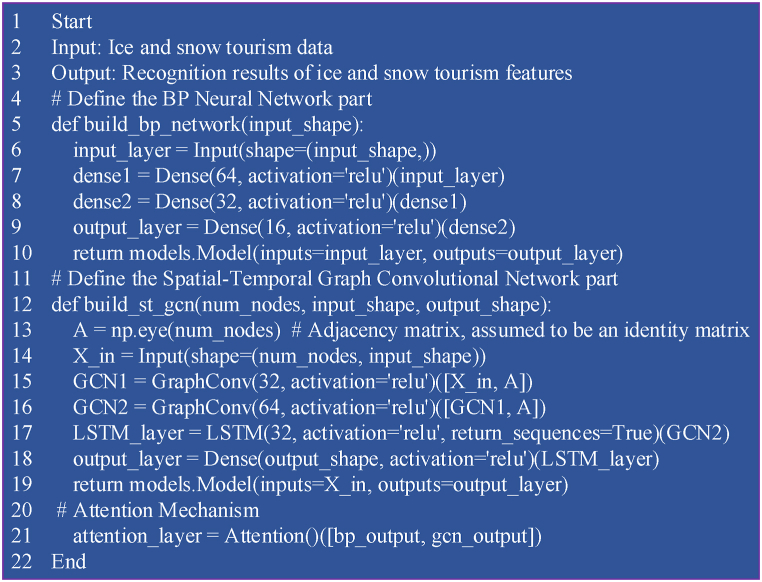
Pseudocode flowchart of BPNN integrated with ST-GCN algorithm applied to IST feature recognition.

Therefore, through multi-level and multi-perspective feature extraction and integration, the model can more accurately identify key features of IST destinations. It provides scientific decision support and development recommendations, promoting the intelligence and sustainable development of IST. Fig. 3 presents the specific pseudocode.

Fig. 3 illustrates the process beginning with the application of web scraping technology to gather data from various online platforms. The collected data undergoes a series of preprocessing steps, including cleaning, standardization, and normalization, to ensure its quality and consistency. Subsequently, the data is partitioned into training and testing sets. The model is initialized by configuring the input and hidden layer nodes of the BPNN, as well as setting the parameters for the temporal window and graph convolution layers of the ST-GCN. During the feature extraction phase, the BPNN performs nonlinear mapping of the input data, adjusting weights and biases via the backpropagation algorithm to achieve rapid convergence. The ST-GCN is then employed to extract spatiotemporal features from the data, while the attention mechanism enhances the recognition of significant information by dynamically adjusting the weights associated with different positions in the sequence.

## 4 Experimental design and performance evaluation

### 4.1 Datasets collection

Data are primarily collected from various online platforms and sources using web scraping technology. This method efficiently gathers diverse types of data, including social media comments, tourist check-in information, weather forecasts, real-time conditions at ski resorts, and consumer records from online booking systems. However, the quality and reliability of the collected data may be influenced by several factors, such as timeliness, completeness, and the diversity of sources. For instance, there may be delays in data updates, incomplete user submissions, or regional and cultural biases in the data. Furthermore, user-generated content can introduce noise, such as irrelevant comments or inaccurate information, potentially affecting data quality and the accuracy of the resulting models.

To ensure data quality and reliability, the experiment adopts various measures, including data preprocessing, validation, and diversity analysis. During data preprocessing, data cleaning is first performed to remove errors, duplicates, or incomplete records from the dataset using automated scripts and manual reviews. This step is crucial for eliminating noise and outliers, as they can distort the training process and prediction results. Next, the data is standardized to convert it into a uniform format or scale, ensuring that data from different sources and types can be compared and analyzed on the same scale. This helps the model more accurately capture data features and patterns. Finally, data normalization is applied by scaling the data to [0, 1] to reduce scale differences between features and avoid overemphasis on certain features during model training. Data validation is conducted through cross-validation with official statistics and other reliable data sources to ensure data consistency. Additionally, the study analyzes the diversity of data sources to ensure that the dataset represents different user groups and regions, thereby reducing bias.

To ensure the effectiveness of model training and testing, the large amount of data collected from multiple online platforms is randomly divided into a training set and a testing set, with 80 % allocated to the training set and 20 % to the testing set. This split ratio aims to provide the model with sufficient data for training while retaining enough independent data to validate the model's predictive ability. To ensure data representativeness, the experiment performs detailed statistical analysis, as shown in Table 1, covering different regions, time periods, and user groups to ensure that the dataset includes key features and trends of IST destinations. Additionally, the study implements stratified sampling to ensure that the proportions of categories in the training set and testing set are consistent with those in the full dataset, avoiding any bias.

**Table 1:** Sample collection statistical results.

Region	Sample Size	Proportion	Average Age	Male Proportion	Female Proportion	Activity Type	Seasonal Peak	User Rating (Average Score)
Northeast China	4210	42 %	32	61 %	39 %	Skiing: 60 %	Winter	4.2
North China	3156	32 %	29	55 %	45 %	Ice Sculpture Viewing: 50 %	Winter	4
Northwest China	1098	11 %	35	53 %	47 %	Ice and Snow Festival: 40 %	Winter	4.1
East China	1536	15 %	28	45 %	55 %	Ice Sports: 30 %	Winter	3.9

### 4.2 Experimental environment

To validate the constructed algorithm, the experiments utilize high-performance hardware and software configurations to ensure efficient and reliable data processing and analysis. The computational power is provided by an Intel Core i9-11900K processor, which supports the execution of complex algorithms. The system is equipped with 64 GB of DDR4 RAM to meet the memory requirements for handling large datasets, thereby avoiding performance bottlenecks. For storage, a 2 TB NVMe SSD is employed, offering rapid data read/write speeds that enhance model training and data retrieval processes. The GPU used is the NVIDIA GeForce RTX 3090, known for its powerful parallel processing capabilities, which are particularly beneficial for computation-intensive tasks such as deep learning. The operating system is Windows 10, chosen for its stability and user-friendly interface. The programming language utilized is Python 3.8, selected for its concise syntax and extensive library support, facilitating rapid development and prototyping. The database management is handled by MySQL 8.0, which efficiently manages and stores large volumes of data. For web scraping, the framework used is Scrapy 2.5.1, a robust tool for data collection. The machine learning frameworks include TensorFlow 2.6.0 and PyTorch 1.9.0, both of which are leading deep learning frameworks with extensive model support and community resources. Data processing is carried out using Pandas 1.3.3 and NumPy 1.21.2, which offer exceptional capabilities for data cleaning, transformation, and analysis. These configurations collectively form a powerful and efficient experimental environment, providing a robust foundation for conducting the research. Table 2 provides details on the experimental environment.

**Table 2:** Experimental environment Configuration.

Environment Type	Configuration Description	Environment Type	Configuration Description
Processor	Intel Core i9-11900K	Operating System	Windows 10
Memory	64 GB DDR4 RAM	Programming Language	Python 3.8
Storage	2 TB NVMe SSD	Database	MySQL 8.0
GPU	NVIDIA GeForce RTX 3090	Web Scraping Framework	Scrapy 2.5.1
Machine Learning Framework	TensorFlow 2.6.0, PyTorch 1.9.0	Data Processing Libraries	Pandas 1.3.3, NumPy 1.21.2

### 4.3 Parameters setting

The specific hyperparameter settings are outlined as follows: For the BPNN, the number of nodes in the input layer corresponds to the dimensionality of the input data. The network includes three hidden layers, configured with 128, 64, and 32 nodes, respectively, and utilizes the ReLU function as the activation function. The number of nodes in the output layer is determined by the requirements of the prediction task. In the ST-GCN model, the time window length is set to 10. The model is comprised of three graph convolutional layers, with convolutional kernel sizes of 64, 128, and 256, respectively. ReLU is employed as the activation function, and a dropout rate of 0.5 is incorporated to prevent overfitting.

Details of these parameter settings are provided in Table 3.

**Table 3:** Experimental parameters.

Parameter type	Value
Number of input layer nodes	6
Number of hidden layer nodes	First layer 128, second layer 64, third layer 32
Activation function	ReLU
Time window length	10
Dropout rate	0.5
Loss function	Cross entropy
Optimizer	Adam
Learning rate	0.001
Training batch size	32
Training cycle	100

### 4.4 Performance evaluation

In order to evaluate the performance of the model constructed, this study conducts comparative experiments from the perspectives of convergence, accuracy, and Root Mean Square Error (RMSE) against the algorithms proposed by ST-GCN [47], BPNN [48], GCN [49], and the model proposed by Movahedi et al. (2023) in related fields.

The convergence value is an indicator used to measure the stability of weight updates during model training. In neural network training, if an algorithm converges quickly to the minimum error, a smaller convergence value indicates a lower loss function value during the training process, which usually suggests better generalization ability. The convergence value is typically represented by the loss function value after the final iteration, as shown in Equation (11).

(11) ConvergenceValue=min(LossFunction)

Recognition accuracy measures the overall classification accuracy, i.e., the proportion of correctly predicted samples, as expressed in Equation (12).

(12) $$Acc=\frac{TP+TN}{TP+FP+TN+FN}$$

*TP* represents the number of true positive samples; *FP* is the number of false positives; *FN* denotes the number of false negatives; and TN indicates the number of true negative samples.

Recall (*Rec*) measures the coverage of positive samples, i.e., the proportion of correctly classified positive samples out of the total number of positive samples, as shown in Equation (13).

(13) $$\text{Re}call=\frac{TP}{TP+FN}$$

Precision (*Pre*) represents the ratio of correctly classified positive instances to the total instances classified as positive, as shown in Equation (14).

(14) $$\mathrm{Pr}\;ecision=\frac{TP}{TP+FP}$$

The F1-score is the weighted harmonic mean of precision and recall, as shown in Equation (15).

(15) $$F1=\frac{2\;\mathrm{Pr}\;ecision\cdot \text{Re}call}{\mathrm{Pr}\;ecision+\text{Re}call}$$

RMSE is a commonly used metric to measure the deviation between the model's predicted values and the actual values. A smaller RMSE indicates lower prediction error and more accurate prediction results, as shown in Equation (16):

(16) $$RMSE=\sqrt{\frac{1}{N}\sum_{t=1}^{N}{\left|x_{i}-y_{i}\right|}^{2}}$$

*N* denotes the number of samples, $x_{i}$ represents the predicted value of the ithi^{th}ith sample, and $y_{i}$ represents the actual value of the *i*th sample.

**Fig. 4:**
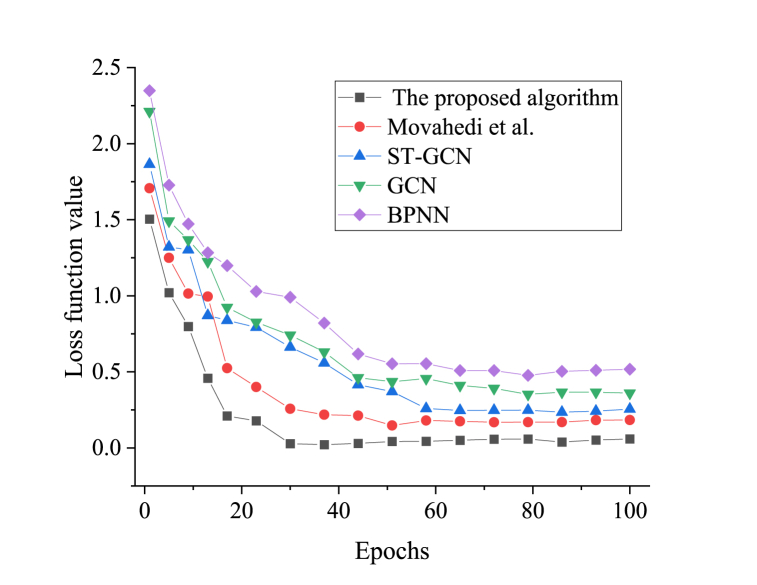
Convergence results of different algorithms.

Firstly, an analysis of the convergence of the proposed algorithm compared to the others is conducted. Fig. 4 displays the results.

Fig. 4 presents the convergence results for each algorithm. As the number of iterations increases, all algorithms demonstrate a converging trend. Notably, the final convergence value of the proposed algorithm is 0.059, which is lower than the convergence values observed in the algorithms based on ST-GCN, GAN, BPNN, and the model proposed by Movahedi et al. (2023). This lower convergence value indicates that the proposed model exhibits superior convergence performance.

**Fig. 5:**
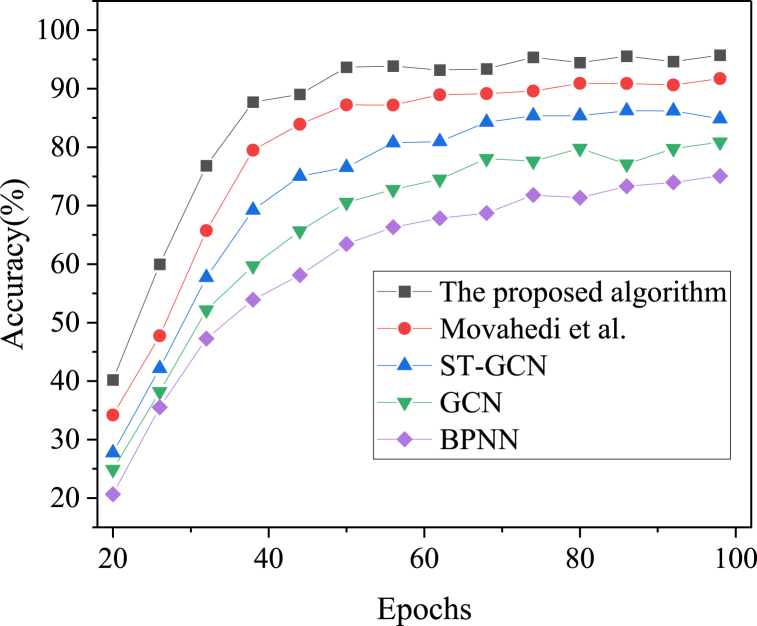
Accuracy results of regional snow and ice tourism prediction under different algorithms over iteration cycles.

**Fig. 6:**
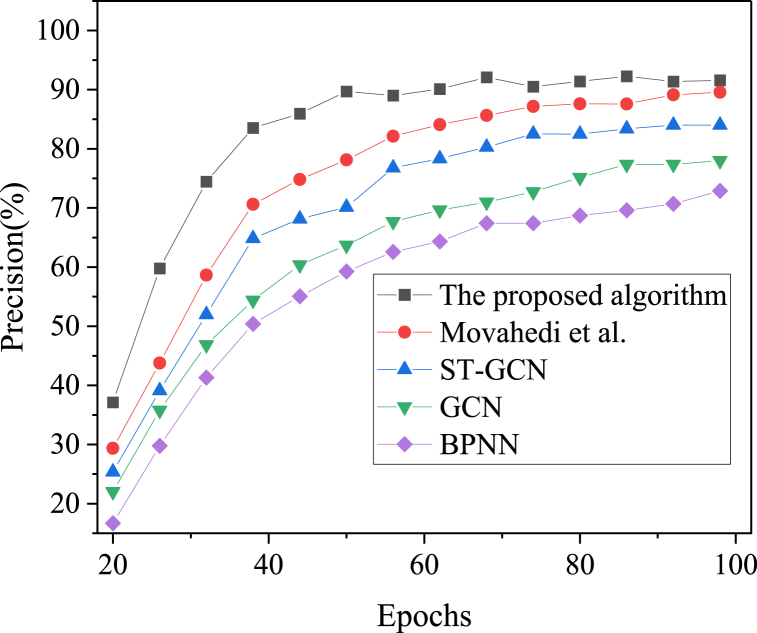
Precision results of regional snow and ice tourism prediction under different algorithms over iteration cycles.

**Fig. 7:**
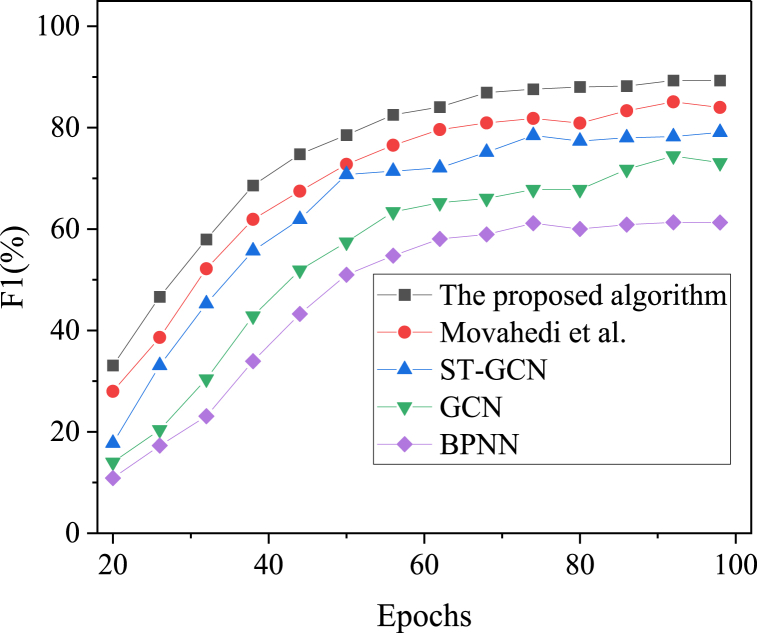
F1 results of regional snow and ice tourism prediction under different algorithms over iteration cycles.

Further analysis of each algorithm's recognition performance is conducted using metrics such as accuracy, recall, and F1-score, as depicted in Fig. 5, Fig. 6, Fig. 7.

Fig. 5, Fig. 6, Fig. 7 present the comparative results of prediction accuracy across various algorithms. As the iteration cycles increase, the accuracy, recall, and F1 scores for each algorithm initially rise and then stabilize. The proposed model achieves an accuracy of 95.74 %, which is at least 4 % higher than the other algorithms. The ranking of prediction accuracy, from highest to lowest, is as follows: the proposed model, the algorithm by Movahedi et al. (2023), ST-GCN, GCN, and BPNN. Further analysis reveals that the recall and F1 scores of the proposed model are 91.57 % and 89.31 %, respectively, at 98 iterations. Thus, the IST feature recognition model, which integrates BPNN with ST-GCN, demonstrates superior performance in predicting the accuracy of regional IST.

**Fig. 8:**
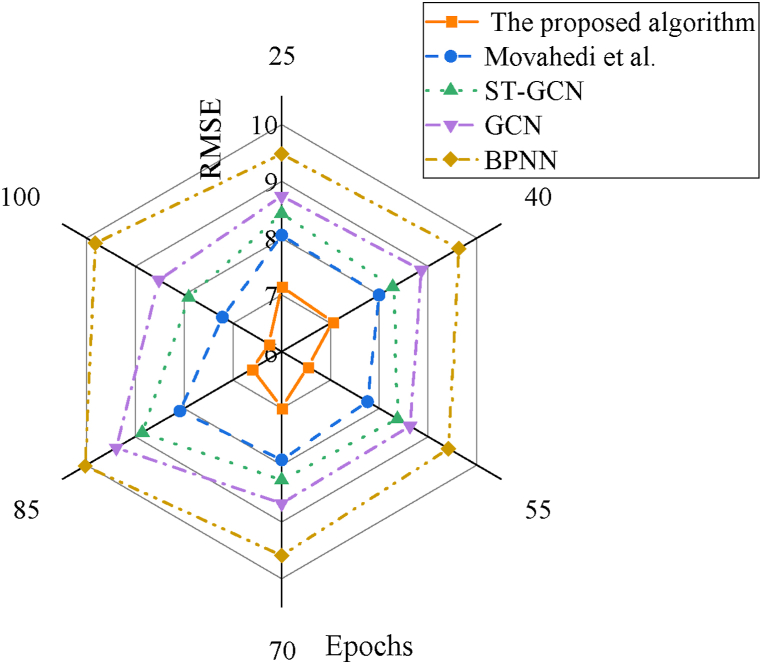
Rmse results of IST prediction under various algorithms.

Further comparisons of RMSE results for IST feature prediction under different algorithms are provided in Fig. 8.

Fig. 8 presents a detailed analysis of the error across different algorithms. As the number of iteration cycles increases, the RMSE of all models generally stabilizes. Notably, the RMSE of the proposed model reaches 6.26 at 100 iterations. In comparison, the prediction errors of other models for IST are significantly higher. This indicates that the IST feature recognition model, which integrates BPNN with ST-GCN, demonstrates superior predictive accuracy with lower errors.

The computational efficiency of each model is compared in Table 4.

**Table 4:** Experimental parameter settings.

Name of the model	Training time (ms)	Prediction time (ms)	Resource consumption (GPU utilization %)
BPNN	131	109	72
GCN	108	95	63
ST-GCN	87	75	56
Movahedi et al. (2023)	73	66	45
The model proposed in this study	62	58	30

Table 4 presents a comparative analysis of the computational efficiency of various models, revealing that the proposed model offers significant advantages in both training and prediction times, as well as resource consumption. Specifically, the proposed model requires only 62 ms for training and 58 ms for prediction, which is substantially lower than the times recorded for other models. For example, the traditional BPNN model takes 131 ms for training and 109 ms for prediction, while the GCN model requires 108 ms for training and 95 ms for prediction. Although the ST-GCN model is slightly faster in training time compared to the GCN, it has a marginally longer prediction time, at 87 ms and 75 ms, respectively. The model proposed by Movahedi et al. (2023), despite its strong performance in training and prediction times (73 ms and 66 ms, respectively), shows less pronounced advantages in resource consumption. The GPU utilization for the proposed model is only 30 %, significantly lower than the BPNN's 72 %, GCN's 63 %, ST-GCN's 56 %, and Movahedi et al.’s 45 %. This indicates that the proposed model not only excels in prediction accuracy but also demonstrates clear advantages in computational efficiency, which is particularly crucial for applications requiring real-time predictions and resource constraints. The efficient computational performance of the model enhances its deployability and applicability, further underscoring the potential and value of this study in practical applications.

To further validate the performance of the proposed model, it was compared with BPNN, Support Vector Machine (SVM), Random Forest (RF), and Gradient Boosting Machine (GBM) algorithms based on prediction accuracy, recall, F1 score, and RMSE, as shown in Table 5.

**Table 5:** Comparison of the proposed algorithm with various machine learning algorithms for identifying the evolution characteristics of snow and ice regions.

Model type	Prediction accuracy	Recall rate	F1 score	RMSE	p value (*t*-test)
BPNN	89.15 %	85.60 %	87.30 %	7.5	<0.05
SVM	88.56 %	84.20 %	86.30 %	7.8	<0.05
RF	91.47 %	88.90 %	87.20 %	6.9	<0.01
GBM	92.01 %	89.40 %	87.70 %	6.7	<0.01
The Proposed model	95.74 %	91.57 %	89.31 %	6.26	<0.01

In Table 5, the proposed model outperforms traditional BPNN and other classic machine learning models across all evaluation metrics. Notably, although the GBM algorithm performs well in predicting the evolution of snow and ice regions, the proposed model still demonstrates a significant advantage in terms of prediction accuracy, recall, and F1 score. These results have been statistically validated through paired t-tests, where the proposed model shows significant performance improvements across all metrics compared to SVM, RF, and GBM.

### 4.5 Discussion

The analysis of experimental results highlights the significant advantages of the proposed IST feature recognition model, which integrates BPNN with ST-GCN, across various dimensions. Firstly, regarding algorithm convergence, all models exhibit convergence as the number of iterations increases. However, the proposed model converges to a value of 0.059, which is markedly lower than the convergence values of models based on ST-GCN, GAN, BPNN, and the model proposed by Movahedi et al. (2023). This suggests that the proposed model outperforms others in terms of convergence, reaching the optimal solution more quickly and stably, in line with the findings of Cao et al. (2023) [50]. In terms of accuracy, the proposed model achieves an accuracy of 95.74 %, reflecting an improvement of at least 4 % over other models. Additionally, at iteration 98, the proposed model attains recall and F1 scores of 91.57 % and 89.31 %, respectively. These metrics collectively demonstrate that the proposed model surpasses other algorithms in the recognition accuracy of regional IST prediction, consistent with the conclusions drawn by Bao et al. (2023) [51] and Zhang et al. (2023) [52].

In practical applications, the proposed BPNN and ST-GCN integrated model provides robust technical support for snow and ice tourism management, offering intelligent management and service solutions for tourist destinations. By accurately predicting tourist flow and behavior patterns, the model enables tourism managers to monitor in real time and issue warnings for potential congestion or safety issues, allowing them to preemptively deploy countermeasures, such as increasing temporary security personnel and adjusting traffic flow, thereby ensuring a safe and comfortable visitor experience. Additionally, the model's in-depth analytical capabilities make personalized tourism recommendations possible. By analyzing tourists' historical behavior and preferences, it can offer customized travel packages and activity suggestions, enhancing visitor satisfaction and loyalty. In terms of resource allocation, the model's predictions of peak periods and visitor preferences assist managers in optimizing resource distribution, such as increasing service staff and maintenance efforts during peak times to improve service quality and operational efficiency. Furthermore, the market trend analysis provided by the model supports the formulation of tourism policies and the development of tourism products, helping businesses stay attuned to market dynamics and make more targeted decisions. For example, if the model identifies an increase in family visitors during a particular period, managers can launch family-friendly activities and packages to meet market demands. These practical applications not only elevate the management level of tourist destinations but also enrich the visitor experience, promoting the sustainable development of the snow and ice tourism industry.

However, applying the BPNN and ST-GCN integrated model to real-world snow and ice tourism management comes with a series of challenges that are crucial for assessing the model's practicality and feasibility. First, the scalability of the model is a key issue, as destinations of different sizes and characteristics may require specific adjustments or retraining of the model. Additionally, the model heavily relies on high-quality and timely data updates, which not only involves the ongoing costs of data collection and integration but also requires an effective data management system to ensure information accuracy and timeliness. Computational resources pose another challenge, as the model demands significant computational power, which might be difficult for small tourism enterprises with limited resources to afford high-performance hardware and specialized software. User acceptance and training are equally important because the model's potential value can only be fully realized when managers and staff can effectively understand and utilize it. Privacy and data security issues must also be addressed, ensuring that relevant laws and regulations are followed during data collection and processing, and that appropriate measures are taken to protect tourists' personal information. Finally, the model's interpretability is crucial for building user trust and providing transparent decision support. Therefore, continuous research and development, along with close collaboration with practitioners, are necessary to ensure the model's effectiveness and feasibility in real-world applications.

Although the feature recognition model for snow and ice tourism, based on the BPNN and ST-GCN algorithms, has demonstrated excellent performance in experiments, it is important to acknowledge potential biases and limitations inherent in any model architecture. As a classical neural network model, BPNN may encounter the “curse of dimensionality” when dealing with high-dimensional data, which can make model training challenging. Additionally, the backpropagation algorithm employed in BPNN may face issues such as vanishing or exploding gradients during training, potentially affecting the model's convergence speed and stability. While ST-GCN is well-suited for handling spatiotemporal data, its dependence on graph structures and time sequences may lead to inconsistent performance across different types of tourism data. The performance of ST-GCN could be compromised if the input data's graph structure changes or if time sequence features are not prominent. Furthermore, the selection and tuning of parameters for ST-GCN can be complex and may require optimization tailored to specific application scenarios.

To ensure the model's scalability and adaptability to different types of tourism data, future research should focus on further evaluating and optimizing the model architecture. Potential solutions to address the limitations of BPNN include introducing regularization techniques, improving network structures, or employing more advanced optimization algorithms. For ST-GCN, designing more flexible graph convolutional layers and time convolutional layers could enhance its adaptability to various graph structures and time sequences.

This study not only addresses gaps in existing research but also provides a scientific foundation and technical support for the management and development of IST destinations. The innovation lies in the integration of BPNN with ST-GCN, which effectively enhances the model's ability to capture and predict spatiotemporal features, resulting in higher prediction accuracy and reduced errors.

## 5 Conclusion

### 5.1 Research contribution

This study presents a feature recognition model for snow and ice tourism by integrating BPNN and ST-GCN. The model not only demonstrates exceptional performance in predicting the evolutionary features and development levels of regional snow and ice tourism destinations, achieving an accuracy rate of 95.74 % and an RMSE of 6.26, but also provides a scientific foundation for the intelligent management of these destinations. This accomplishment enhances the management efficiency and visitor experience of snow and ice tourism destinations and offers valuable technical insights for the planning and management of other tourism sites. Furthermore, the application of this model highlights the potential of deep learning technology in the tourism sector, offering new directions for future research and practice.

### 5.2 Future works and research limitations

Despite the significant achievements in snow and ice tourism feature recognition, several limitations remain. First, data collection primarily relies on web scraping technology, which may be influenced by the timeliness and authenticity of online data. To enhance the model's robustness and generalizability, future research should incorporate more diverse and reliable data sources, such as social media reviews, visitor physiological data, and real-time monitoring data from tourism destinations. Second, the model's generalizability and applicability to other tourism destinations have not been thoroughly validated. Future studies should apply the model to various types of tourism destinations and evaluate its performance across different environments and conditions through practical testing and validation. Moreover, to improve the model's practicality and adaptability, future research could investigate more efficient algorithms and computational methods to reduce the model's computational complexity and enhance its real-time response capabilities. Establishing user interaction and feedback mechanisms, collecting visitor feedback and suggestions, and continually optimizing the model's predictions and user experience will further increase its practicality and user satisfaction. These targeted improvements can elevate the model's application value, providing stronger support for the intelligent development and management of tourism destinations.

## Data availability statement

Data will be made available on request.

## Declaration of competing interest

The authors declare that they have no known competing financial interests or personal relationships that could have appeared to influence the work reported in this paper.
